# Increasing photoperiod stimulates the initiation of spring migratory behaviour and physiology in a facultative migrant, the pine siskin

**DOI:** 10.1098/rsos.180876

**Published:** 2018-08-08

**Authors:** Ashley R. Robart, Mali M. K. McGuire, Heather E. Watts

**Affiliations:** 1 School of Biological Sciences, Washington State University, Pullman, WA 99164, USA; 2 Center for Reproductive Biology, Washington State University, Pullman, WA 99164, USA; 3 Department of Biology, Loyola Marymount University, Los Angeles, CA 90045, USA

**Keywords:** bird, facultative migration, photoperiod, migratory state, migratory restlessness, testosterone

## Abstract

The transition to a migratory state involves coordinated changes in physiology and behaviour. In species with regular, predictable (obligate) migrations, increasing day length triggers the expression of a spring migratory state and androgens play an important role in stimulating its development. By contrast, we know little about the environmental cues and endocrine mechanisms that regulate migration in species with less predictable (facultative) migrations. Here, we tested whether photoperiod stimulates a migratory state in a facultative nomadic migrant, the pine siskin (*Spinus pinus*). We exposed wintering birds to either a naturally increasing or short-day photoperiod and measured physiological and behavioural changes indicative of a migratory state. We also examined changes in circulating hormones that may play a role in the migratory transition. Natural-day, but not short-day, birds displayed physiological preparations for migration, including increases in fat deposition, and showed increased levels of migratory restlessness. We found no evidence for a role of corticosterone in the migratory transition, but testosterone may be important. This study is the first experimental test of the role of photoperiod in regulating facultative migration and demonstrates that the predictive cue used by many obligate migrants to time spring migration is also important in a facultative migrant.

## 1. Introduction

Each spring obligate migrants undertake movements from wintering areas to breeding grounds. The timing, direction and distance of movements are consistent, predictable and often have a genetic basis [1,2]. The regularity of these obligate migrations allows individuals weeks or months to make physiological and behavioural preparations for a spring migratory state [3]. For obligate migratory birds, increasing day length acts as a stimulatory cue for migratory preparations, including increases in body mass, fat deposition, muscle hypertrophy and increased locomotor activity in captive birds [1,3]. Nocturnal activity, termed migratory restlessness or *Zugunruhe*, in particular has been interpreted as an indicator of behavioural readiness to migrate [4,5]. Increasing day length also triggers an increase in androgen production, which is thought to induce the physiological and behavioural changes associated with transitioning to a migratory state [6–10]. Other endocrine mechanisms, including corticosterone and thyroid hormone signalling, may also play a role in the transition [11–16]. For example, elevated corticosterone levels may facilitate the metabolic demands associated with the increased energy expenditure experienced during migratory flight [17,18] and may act as a stimulus for migratory activity [19–21]. Migratory preparedness can influence both survival [22] and reproduction [23], underscoring the critical importance of the physiological and behavioural changes that occur in the anticipation of migration.

However, not all migratory patterns are as consistent and predictable as obligate migration. Migratory movements occur along a spectrum, with facultative migration, characterized by irregular movements that vary in their direction, timing and distance, at the other end of the continuum [2,24]. Much less is known about facultative migration than obligate migration, likely due in part to the challenges of studying it in the field. Facultative migrants typically use food resources that are highly variable in their abundance and distribution, such as rodents and conifer seeds [2], and facultative migratory patterns are suggested to have evolved in response to the unpredictability of resources [2,24]. The timing of facultative migrations is thought to be driven by local environmental conditions [24–29], but it is unclear whether facultative migrants also use day length as an initial predictive cue to initiate migration. Given the high degree of spatial and temporal variability in facultative movements, the utility of such a predictive cue may be reduced. Nonetheless, several bird species that make nomadic migrations, a type of facultative migration, display migratory preparations such as fat deposition and behavioural readiness (i.e. increased locomotor activity) in the spring, similar to obligate migrants [30–33]. However, it is unknown whether these changes in nomadic migrants are, in fact, a response to increasing day length. An alternative hypothesis is that these changes reflect an endogenous circannual rhythm rather than a response to photoperiod [34]. If these physiological and behavioural changes are a response to photoperiod, it may be that nomadic migrants use this initial cue to generate a window of ‘readiness’ to migrate, with additional information about local environmental conditions determining whether departure occurs [24,25].

This study aimed to distinguish between alternative hypotheses that initiation of a spring migratory state is driven by increasing day length versus a circannual rhythm in a facultative nomadic migrant, the pine siskin (*Spinus pinus*). Pine siskins are a small North American finch that displays low site fidelity [35,36]. Movements are nomadic, lacking consistent directional orientations and precise timing, and irruptive, with large flocks migrating to areas outside their typical range in some years [2,27,37]. Pine siskins are most frequently observed moving in the spring and autumn [35,36]. Autumn movements are thought to be tightly linked to the availability of an important food source, conifer seeds [27,38], whereas spring migratory movements may be necessary to return individuals to suitable breeding areas, particularly in irruptive years [25,30]. Additionally, pine siskins are one of the species of nomadic migrants that show a spring migratory transition in captivity [32].

To distinguish between the alternative hypotheses, we used an experimental approach, exposing birds to either a photoperiod that mimicked naturally increasing day length or a constant, short-day photoperiod and measured physiological and behavioural changes that are indicators of a migratory state. Expression of a migratory state in birds on increasing day length, but not short days, is predicted if the migratory transition is driven by the photoperiod. Expression of a migratory state in both groups is predicted if the transition is driven by a circannual rhythm. A second goal of this study was to investigate the potential role of changes in circulating testosterone and corticosterone in the transition to a migratory state in a facultative nomadic migrant. Both hormones have been implicated in the transition to a spring migratory state in obligate migrants, and corticosterone has also been proposed as an endocrine mechanism triggering facultative migration [11,25,39]. Therefore, we quantified these hormones during the photoperiod manipulation experiment, and we conducted a second experiment examining, in greater detail, the relative timing of changes in circulating testosterone and migratory physiology and behaviour. By examining whether the endocrine mechanisms and a stimulatory cue that triggers a spring migratory state in obligate migrants are also involved in regulating facultative migration, we aim to understand both the potential similarities, as well as differences, between birds whose movement patterns represent opposite ends of the migratory spectrum.

## 2. Material and methods

### 2.1. Experimental design overview

Our experiment contained two phases: an experimental manipulation of photoperiod (Photoperiod experiment) and a fine-scale sampling of circulating testosterone levels (Timing experiment). The Photoperiod experiment was conducted from 22 December 2015 to 7 June 2016. The Timing experiment occurred from 24 February to 14 April 2017. Experiments focused primarily on males as subjects to ensure sufficient sample sizes given expected sex differences, primarily in testosterone levels. However, we also included as many females as was logistically feasible for each experiment. Further details on experimental design for each phase are provided below.

Birds for both experiments came from capture sites in California, Oregon, Washington and Wyoming, USA (see electronic supplementary material for site details). Birds were collected under all necessary permits and permissions (details in the ‘Ethics’ section). After capture, birds were brought to Loyola Marymount University in Los Angeles, CA and housed indoors in small groups on a photoperiod that simulated natural changes in day length locally (34° N latitude). Birds were provided with ad libitum water and grit, as well as Roudybush Small Bird Maintenance Diet (Woodland, CA) and a nyjer thistle and sunflower heart seed mixture. For both phases of the study, birds were placed in individual cages (approx. 34 cm (width) × 38.5 cm (length) × 43 cm (height)) at least one week prior to the start of data collection.

### 2.2. Photoperiod experiment

#### 2.2.1. Overview

Birds were held on either a photoperiod that mimicked naturally changing day length (hereafter ‘natural-day’; *N* = 14 males, 7 females) at 34° N latitude or on a 10 L : 14 D photoperiod, which mimicked day length on the winter solstice (hereafter ‘short-day’; *N* = 13 males, 7 females) at this latitude. Capture site and age were balanced across the treatment groups, and birds had auditory but not visual access to others in the same photoperiod treatment. At the end of the experiment, the short-day birds were photostimulated (15 L : 9 D); once all birds initiated postnuptial moult, they were returned to a photoperiod that simulated naturally changing day length (34° N latitude).

#### 2.2.2. Body measurements and blood collection

Body measurements were made every two weeks; body mass, furcular and abdominal fat deposits, and flight (pectoralis) muscle size and colour were recorded. Measurements were conducted between 10.00 and 15.00. Birds were measured in the same order each day to hold individual sampling time consistent across the experiment. Body mass was weighed to 0.01 g on an electronic balance. Furcular and abdominal fat were scored visually on a scale from 0 (no fat) to 5 (bulging fat) [40]. Flight muscle size was scored visually on a scale of 0 (muscle concave with keel very prominent) to 3 (muscle bulging over keel) [41]. Changes in flight muscle colour have also been observed during the transition to a migratory state, with lighter coloration indicating muscle that has a higher lipid content (M. Ramenofsky 2017, personal communication). Flight muscle coloration was quantified using digital photographs and the Image Calibration and Analysis Toolbox [42] for ImageJ (see the electronic supplementary material for full protocol).

We collected blood samples from males once a month over an approximately 5-day period. Blood was collected from the alar vein into heparinized microhematocrit tubes. Blood collection occurred approximately 2 h after lights on for each photoperiod treatment group. To capture baseline corticosterone levels, blood samples used to measure corticosterone were collected within 3 min of entering a room that day [43]. Samples used to measure plasma testosterone were collected within 10 min. Samples were stored on ice until centrifugation at 13 000*g* for 9 min. We measured haematocrit for the first tube of blood and collected the plasma. Plasma was stored at −20°C until assayed for corticosterone and testosterone; the January and May samples were used for a separate study.

#### 2.2.3. Activity data

We recorded activity using a passive infrared sensor (Starr Life Sciences Corp., Oakmont, PA) placed on the top of each cage. Sensors were attached to a VitalView Data Acquisition System (Starr Life Sciences Corp., Oakmont, PA). We continuously recorded movement and summed activity in 10 min intervals. We calculated mean diurnal and nocturnal activity for a 5-day period (when birds were not handled for body measurements or blood collection) every two weeks. Mean diurnal activity was calculated by averaging the 10 min intervals in which the lights within each treatment room were on. Intervals during which the lights transitioned from on to off and vice versa were excluded, as well as intervals in which researchers were present in the room. Pine siskins exhibit a 3 to 4 h period of quiescence after lights off, with peak nocturnal activity occurring after 23.00 [32]. We therefore calculated nocturnal activity by averaging the 10 min intervals between 23.00 and 03.00. We conservatively did not include activity after 03.00 as pine siskins display pre-dawn activity similar to other wintering birds, which is believed to be distinct from migratory restlessness [12,32,44].

#### 2.2.4. Hormone assays

We used enzyme immunoassay kits from Enzo Life Sciences to measure plasma corticosterone (ADI-901-097) and testosterone (ADI-901-065). Samples were run in duplicate with plasma dilutions and steroid displacement buffer concentrations optimized for pine siskins as described previously [45]. Plasma pools were run as standards on all plates to estimate coefficients of variation (CVs), and detection limits were calculated for each plate as the upper limit of the 95% confidence interval for the blank wells. For all assays, samples below the detection limit were assigned the plate-specific detection limit as their value. All samples for an individual were run on the same plate, with photoperiod treatment randomized across plates. For corticosterone, samples were run in a 1 : 20 dilution with 1% (of raw plasma volume) steroid displacement buffer with a six-point standard curve ranging from 2000 to 1.95 pg ml^−1^. Samples were run across three plates; the intra-assay CV was 10.9% and the inter-plate CV was 14.2%. The mean detection limit was 0.52 ng ml^−1^; 47.2% of samples were below the detection limit of their plate. For testosterone, samples were run in a 1 : 20 dilution with 0.5% steroid displacement buffer. Samples were run using four plates; the intra-assay coefficient of variation was 18.2% and the inter-plate coefficient of variation was 4.5%. The mean detection limit was 0.22 ng ml^−1^; 9.8% of samples were below the detection limit of their plate.

### 2.3. Timing experiment

#### 2.3.1. Overview

We examined, in further detail, the relationship between changes in testosterone and migratory behaviour and physiology using an experiment with a more intensive sampling protocol. Birds (*N* *=* 16 males, 12 females) were housed on a photoperiod that mimicked naturally changing day length at 42° N latitude. Birds were housed on this latitude for approximately three months prior to the start of data collection. Although this is a different latitude from the Photoperiod experiment, this latitude is more representative of locations where pine siskins can be found in both the winter and the summer. Thus, using this second latitude afforded the opportunity to assess whether the effect of day length on a spring migratory state is robust across more than one latitude [46].

#### 2.3.2. Body measurements and blood collection

We measured body condition and collected blood from each bird every two weeks. However, each week we only sampled half the birds. This allowed more frequent sampling while minimizing the amount of blood collected from individual birds. Sampling typically spanned 2–3 days each week. Blood collection, including time of day, and sample processing were conducted in the same manner as the Photoperiod experiment. Blood samples were collected within 5 min of entering the room for the day. We measured body condition between 09.00 and 11.00 on the morning that we collected a blood sample using the same parameters as during the Photoperiod experiment, except that muscle colour was not included. We also measured cloacal protuberance (CP) size in males, an androgen-dependent trait, as an additional indicator of androgen activity [47,48]. Specifically, we measured the length of the CP (to the nearest 0.1 mm) from the base at the abdomen to tip using dial callipers.

#### 2.3.3. Activity data

We recorded activity in an identical manner as during the Photoperiod experiment. As we were interested primarily in the effect of testosterone on migratory restlessness, we only calculated mean nocturnal activity. We calculated each bird's mean activity per 10 min interval between 23.00 and 03.00 for the two nights prior to when it was sampled for blood and body condition.

#### 2.3.4. Hormone assays

Plasma testosterone was assayed in an identical manner to the Photoperiod experiment testosterone samples. Samples were run across four plates; the intra-assay CV was 14.9% and the inter-plate CV was 12.4%. The mean detection limit was 0.13 ng ml^−1^; 5.4% of samples were below the detection limit of their plate.

#### 2.3.5. Statistical analyses

We used linear mixed models (LMMs) and generalized linear mixed models (GLMMs) to investigate effects of photoperiod on migratory physiology and behaviour. We used R v. 3.2.4 [49] and the lme4 package [50] for all mixed models, except that nocturnal activity was analysed using the glmmADMB package [51]. For the Photoperiod experiment, models tested the effects of time (days since winter solstice), photoperiod treatment and the interaction between time and treatment on response variables. Models for the Timing experiment included the effects of time (days since winter solstice), sex and the interaction between time and sex, with the exception of the LMM for CP length, which only included time. In all analyses, individual identity was included as a random effect (random intercepts). We used likelihood-ratio tests and Wald tests for the LMMs and GLMMs, respectively, to test model effects. We visually inspected residual plots to check for deviations from normality and homoscedasticity. We used principal component analysis (PCA) to create a single measure of body condition that included mass and total fat (see electronic supplementary material) and analysed body condition for the Photoperiod and Timing experiments separately. As we were most interested in changes in muscle size that indicated muscle hypertrophy (size of 3), we focused our analysis of muscle size on the transition between sizes 2 and 3. Pine siskins kept under laboratory conditions with ad libitum access to food typically have muscle profiles larger than 1 (A.R.R. 2015, personal observation). Thus, we confined our analysis to instances of birds with muscle sizes of 2 or 3 (93.4% and 88.1% of Photoperiod and Timing experiment measurements, respectively) and used a GLMM with a binomial distribution and log link for muscle size. We used PCA to create a PC that quantified changes in muscle colour using digital photographs (see the electronic supplementary material). The flight muscle colour PC did not meet assumptions of normality and was log transformed prior to analysis. Nocturnal activity for both experiments was over-dispersed and zero-inflated; we therefore used the negative binomial (nbinom1) distribution in the glmmADMB package. For the Timing experiment, there was one bird that was an outlier with respect to activity levels; the inclusion of this bird did not qualitatively change the results of the analysis and it was left in the final analysis. Diurnal activity and haematocrit were only analysed for the Photoperiod experiment and were normally distributed. We log transformed corticosterone prior to analysis. Given the large number of samples below the detection limit for the corticosterone assay, we also used a GLMM with binomial distribution and log link to analyse whether the proportion of samples below the detection limit differed between the photoperiod treatments. Testosterone was not normally distributed for either dataset; for the Photoperiod experiment, we performed a Box–Cox transformation (*λ* = −0.15) and used a log transformation for the Timing experiment.

To investigate the timing of changes in physiology and behaviour, we used the changepoint package [52] in R. We used the ‘CUSUM’ distribution, which does not assume a normal distribution, and specified a single change point. For analyses with a significant treatment × time or treatment effect, we analysed each group (e.g. natural versus short, male versus female) separately to find the change point for each. For analyses that indicated only a significant effect of time, both groups were analysed together to find a single change point.

## 3. Results

### 3.1. Photoperiod experiment

**Figure 1.**
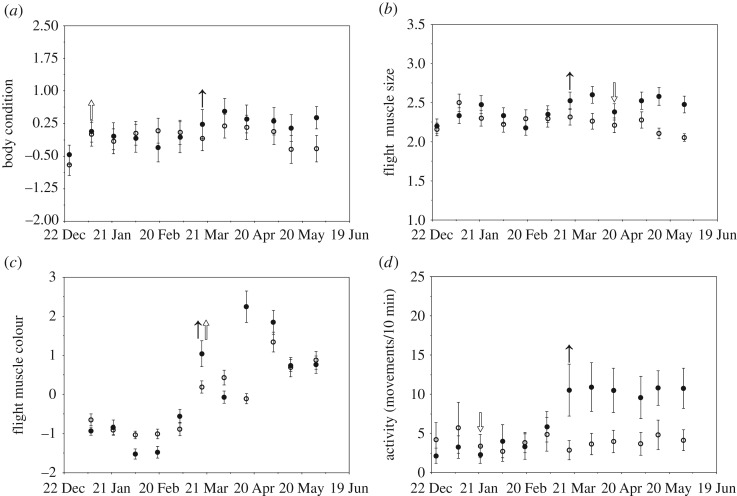
Body condition (*a*), flight muscle size (*b*), flight muscle colour (*c*) and nocturnal activity (*d*) of natural-day (filled circles and arrows) and short-day (open circles and arrows) birds during the Photoperiod experiment. See electronic supplementary material, tables S1 and S3, for PC loading values for muscle colour and body condition, respectively. Arrows indicate significant increases or decreases in mean trait value, as indicated by change point analysis. Data points are means ± 1 s.e.m.

**Figure 2.**
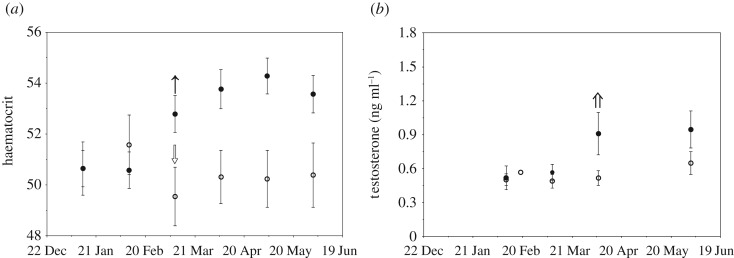
Haematocrit (*a*) and testosterone (*b*) of natural-day (filled circles and arrow) and short-day (open circles and arrow) birds during the Photoperiod experiment. Arrows indicate significant increases or decreases in mean trait value, as indicated by change point analysis. Testosterone did not differ between natural-day and short-day birds and the two treatment groups were analysed together for the change point analysis (double arrow). Data points are means ± 1 s.e.m.

The PC representing body condition explained 93.0% of the variation in mass and total fat and loaded positively for both measures (see electronic supplementary material, table S3). There was a significant time × treatment effect on body condition (time × treatment: $\chi_{1}^{2}=4.73,$ *p* = 0.03; time: $\chi_{1}^{2}=25.44,$ *p* < 0.0001; treatment: $\chi_{1}^{2}=0.007,$ *p* = 0.93), with natural-day birds showing a greater increase in body condition across the experiment compared with short-day birds (figure 1*a*). Muscle size was significantly affected by the interaction between time and treatment (time × treatment: *z* = −19.40, *p* < 0.0001; time: *z* = 11.59, *p* < 0.0001; treatment: *z* = 1.22, *p* = 0.22); muscle size increased during the experiment for natural-day birds, but decreased in the short-day birds (figure 1*b*). There was a significant time × treatment effect on muscle colour (time × treatment: $\chi_{1}^{2}=6.32,$ *p* = 0.02; time: $\chi_{1}^{2}=228.65,$ *p* < 0.0001; treatment: $\chi_{1}^{2}=0.43,$ *p* = 0.51). Muscle colour scores increased for both short- and natural-day birds, indicating a lightening of the muscle (see electronic supplementary material, table S1), but increased to a greater degree for the natural-day birds (figure 1*c*). Natural-day birds increased their nocturnal activity compared with short-day birds (figure 1*d*), and the interaction between time and treatment significantly affected nocturnal activity (time × treatment: *z* = −5.42, *p* < 0.0001; time: *z* = 8.56, *p* < 0.0001; treatment: *z* = 0.85, *p* = 0.39). There was also a significant time × treatment effect on diurnal activity (time × treatment: $\chi_{1}^{2}=8.63,$ *p* = 0.003; time: $\chi_{1}^{2}=44.55,$ *p* < 0.0001; treatment: $\chi_{1}^{2}<0.0001,$ *p* = 0.99), with diurnal activity decreasing initially for natural-day birds, followed by a later decline in diurnal activity in short-day birds (see electronic supplementary material, figure S3). There was a significant time × treatment effect (time × treatment: $\chi_{1}^{2}=17.79,$ *p* < 0.0001; time: $\chi_{1}^{2}=8.38,$ *p* = 0.004; treatment: $\chi_{1}^{2}=3.95,$ *p* = 0.05) on haematocrit, with natural-day birds increasing haematocrit levels, while the short-day birds experienced a decrease in haematocrit during the experiment (figure 2*a*). Testosterone varied significantly during the experiment (time: $\chi_{1}^{2}=9.90,$ *p* = 0.002). The natural-day birds increased testosterone earlier and had higher levels than the short-day birds (figure 2*b*), but this pattern was not statistically significant (treatment: $\chi_{1}^{2}=1.65,$ *p* = 0.20; time × treatment: $\chi_{1}^{2}=2.26,$ *p* = 0.13). Corticosterone levels remained low in both natural-day (0.90 ± 0.09 s.e.m. ng ml^−1^) and short-day (0.82 ± 0.06 s.e.m. ng ml^−1^) birds (treatment: $\chi_{1}^{2}=0.07,$ *p* = 0.79) for the duration of the experiment (time: $\chi_{1}^{2}=0.96,$ *p* = 0.33; time × treatment: $\chi_{1}^{2}=1.73,$ *p* = 0.19; see electronic supplementary material, figure S4). The proportion of samples below the detection limit for the corticosterone assay did not differ between photoperiod treatments (*z* = −1.17, *p* = 0.24).

The change point analysis indicated that body condition (figure 1*a*), muscle size (figure 1*b*), muscle colour (figure 1*c*) and nocturnal (figure 1*d*) and diurnal activity (electronic supplementary material, figure S3) all had a significant change in their mean value for the natural-day birds around 21 March, while haematocrit (figure 2*a*) increased prior to these changes, at the beginning of March. As there was not a significant treatment effect on testosterone, we analysed both the short-day and natural-day birds together for the change point analysis, which indicated testosterone increased at the beginning of April (figure 2*b*).

### 3.2. Timing experiment

**Figure 3.**
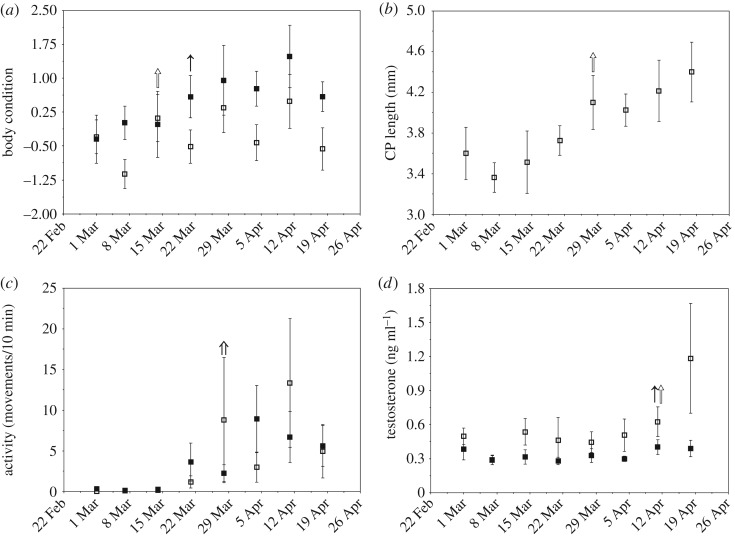
Body condition (*a*), cloacal protuberance (CP) length (*b*), nocturnal activity (*c*) and testosterone (*d*) in males (open squares and arrows) and females (filled squares and arrows) during the Timing experiment. See electronic supplementary material, table S3, for PC loading values for body condition. Arrows indicate significant increases or decreases in mean trait value, as indicated by change point analysis. Nocturnal activity did not differ between males and females and the sexes were analysed together for the change point analysis (double arrow). Data points are means ± 1 s.e.m.

The PC representing body condition for this experiment explained 94.7% of the variation in mass and total fat and loaded positively for both mass and total fat (see electronic supplementary material, table S3). There was a significant time × sex effect on body condition (time × sex: $\chi_{1}^{2}=5.10,$ *p* = 0.02; time: $\chi_{1}^{2}=35.33,$ *p* < 0.0001; sex:$\;\chi_{1}^{2}=2.46,$ *p* = 0.12), with males increasing in body condition shortly before females (figure 3*a*). There was a trend for flight muscle size to increase over the duration of the study (time: *z* = 1.85, *p* = 0.06); however, neither sex (*z* = −1.13, *p* = 0.26) nor the interaction between sex and time (*z* = 1.12, *p* = 0.26) significantly predicted flight muscle size. Males had a significant increase in CP length during the experiment (time: $\chi_{1}^{2}=29.63,$ *p* < 0.0001; figure 3*b*). Nocturnal activity increased significantly during the experiment (time: *z* = 3.65, *p* = 0.0003), and both sexes responded similarly, as there was neither a sex (*z* = −0.78, *p* = 0.44) nor sex × time effect (*z* = 0.55, *p* = 0.58; figure 3*c*) on nocturnal activity. Males had significantly higher testosterone levels than females (sex: $\chi_{1}^{2}=3.98,$ *p* = 0.04), and testosterone increased significantly (time: $\chi_{1}^{2}=9.47,$ *p* = 0.002) during the experiment, although there was no significant sex × time interaction on testosterone ($\chi_{1}^{2}=1.90,$ *p* = 0.17; figure 3*d*).

The change point analysis indicated that the increases in body condition (figure 3*a*) for both sexes occurred prior to the increase in nocturnal activity (figure 3*c*). CP length in males increased (figure 3*b*) at the same time as activity increased, approximately 29 March; however, testosterone did not increase until mid-April in each sex (figure 3*d*).

## 4. Discussion

This study indicates that photoperiod is an important cue regulating the transition to a spring migratory state in pine siskins, a facultative nomadic migrant. Birds exposed to a naturally increasing day length showed changes in body condition, flight muscle size, haematocrit and nocturnal activity that were consistent with initiating a migratory state [1]. These changes contrasted with the birds held on a constant short-day photoperiod, which only displayed a lightening of flight muscle coloration. The lack of migratory preparations in the short-day birds indicates that a spring migratory state in pine siskins is driven by changes in day length rather than by an endogenous circannual rhythm. Intensive sampling during the Timing experiment revealed that body condition increased prior to the increase in migratory restlessness. This suggests that photoperiod is an initial predictive cue for spring migration in pine siskins, with birds preparing physiologically before displaying migratory behaviour. These results represent the first experimental test of the role of photoperiod in regulating facultative migration and suggest that the predictive cue used by many obligate migrants to time their spring migratory movements [1,3] may be used by birds across a broader range of the migratory spectrum [2,24].

The physiological preparations observed in the natural-day birds are likely to support increased energetic demands associated with migratory flight. In both experiments, these birds displayed significant increases in body condition, indicating increases in body mass and fat. These changes in body condition mirror the physiological changes displayed by obligate migrants during spring migratory preparations [1,15,53,54], with stored fat serving as the main fuel during migratory flight [55,56]. Obligate migrants also cope with the increased energetic demands of migratory flight by increasing haematocrit levels, which increases the oxygen carrying capacity of the blood [57,58]. Here, we found a significant increase in haematocrit in natural-day birds, occurring shortly before they increased nocturnal activity. Natural-day birds also had a significant increase in flight muscle size, again consistent with migratory preparations [1,53]. Finally, the observed changes in flight muscle coloration were somewhat surprising. The muscle colour lightened in both the natural-day and short-day birds. This change happened during the sampling period that body condition and nocturnal activity increased in only the natural-day birds. The lightening is believed to result from greater reflectance of the muscle as lipid pools, which support energy production within the tissue, are deposited in the flight muscle fibres (M. Ramenofsky 2017, personal communication). This pattern suggests a decoupling from the other physiological preparations for migration, because it occurred in both treatment groups, whereas other physiological preparations were only observed in the natural-day treatment. However, additional research is needed to better understand the physiological mechanism(s) underlying lightening of the flight muscle colour, and how it relates to other migratory preparations.

One of the most widely recognized indicators of a migratory state in captive studies is the expression of nocturnal migratory restlessness, or *Zugunruhe* [3]. During both experiments, natural-day birds had a significant increase in nocturnal activity. The two experiments were conducted at different latitudes, suggesting increasing day length induces a migratory state in pine siskins across photoperiodic conditions. The timing of the initiation of nocturnal activity was relatively consistent between the two experiments and occurred approximately once the 12 L : 12 D threshold had been crossed. This has also been found to be an important threshold for expression of a migratory state in some obligate migrants [15,18]. The intensive sampling during the Timing experiment revealed that males and females increased their nocturnal activity the same week. The nomadic nature of facultative migration means that individuals often breed in different locations each year. The synchronization of both timing of departure, as well as movements once migration is underway, would ensure that both sexes arrive together in breeding areas. During the period when nocturnal activity significantly increased, we observed a significant decline in diurnal activity in the natural-day birds. Previous work on pine siskins has shown that a decline in diurnal activity, associated with an increase in migratory restlessness, is primarily driven by a reduction in afternoon activity [32]. Reduced afternoon activity may facilitate acquiring orientation information or allow for digestion before nocturnal departure [44] and has been observed in other birds with nocturnal migration [44,59].

Increases in circulating corticosterone are hypothesized to be particularly important in initiating facultative migration [11,25,60]. However, neither the natural-day nor short-day birds experienced a significant change in corticosterone during the experiment. Thus, we find no evidence for a role of corticosterone in the transition to a spring migratory state in pine siskins. This is in contrast to a number of studies from obligate migrants in which elevated diurnal corticosterone levels correspond to the expression of migratory restlessness in captive birds and migratory departures in free-living birds [15,19,20]. We caution, however, that interpretation of our results may be limited as blood samples were collected during morning hours, not during the night. In at least two species of obligate migrants, corticosterone levels during the dark phase show the strongest relationship with nocturnal migratory restlessness [16,53,54]. Thus, it remains to be determined whether there is a relationship between corticosterone levels during the dark phase and migratory restlessness in pine siskins.

The endocrine mechanism most closely associated with a spring migratory state in obligate migrants is an increase in circulating androgen levels. It is believed that an initial increase in testosterone, in response to increasing day length, leads to changes in body composition (e.g. increased fat deposition and muscle hypertrophy), as well as nocturnal activity [6,7,10]. The results of these two experiments do not provide compelling evidence that an increase in circulating testosterone is a key mechanism driving the initiation of a spring migratory state in pine siskins, though we cannot rule out an important role for testosterone. There was a trend for the natural-day birds to show an increase in testosterone in April compared with the short-day birds, but this difference was not statistically significant. During the Timing experiment, there was a significant increase in testosterone, but it occurred several weeks after both body condition and nocturnal activity increased. We did, however, observe a significant increase in CP length in males prior to the increase in circulating testosterone levels, and this increase occurred during the same sampling period that migratory restlessness increased significantly. As CP size is androgen-dependent [47,48], this suggests effects of androgens on a time scale appropriate for the migratory transition. Hormone manipulation experiments have provided strong evidence for the role of testosterone in initiating a spring migratory state [7–10], though details about the quantity and timing of androgen secretion necessary to activate migratory physiology and behaviour remain unresolved [61]. In particular, obligate migrants may express a migratory state in spring when circulating levels of androgens are low [62,63], including in species where testosterone has been shown to be important in stimulating migratory physiology and/or behaviour (see control groups in [8,9]). These results suggest that low levels of androgens or a pulse of androgen earlier in the year may be sufficient to initiate spring migratory preparations. Thus, we cannot rule out the possibility that small, though not statistically significant, increases in testosterone such as we observed or a brief pulse of androgen, not captured in our sampling, may play a role in stimulating spring migration. Finally, it is also possible that changes in photoperiod influence the migratory transition via testosterone signalling by altering responsiveness to testosterone. For example, this could involve changes in the expression of androgen receptors, oestrogen receptors and/or aromatase activity, as it is currently unknown whether effects of testosterone in this context are mediated by activation of androgen or oestrogen receptors (following aromatization of androgens to oestrogens). Androgen and oestrogen receptors, as well as aromatase, show seasonal changes in expression in songbirds [64–66], although little is currently known about these changes in relation to migratory transitions.

This study demonstrates that increasing day length alone is sufficient to stimulate both physiological changes that occur in anticipation of spring migration as well as the subsequent expression of migratory restlessness in a facultative migrant. It remains to be determined how the spring migratory state observed in captive pine siskins is expressed in free-living birds. Free-living birds may exhibit migratory preparations in response to increasing photoperiod, but the initiation of migration (i.e. migratory departure) may depend on other local environmental cues, such that migration may not always occur [30]. Alternatively, increasing photoperiod may stimulate migratory preparations as well as departure, with local cues influencing termination of migration such that immediate settlement may occur when conditions are favourable [25,67]. Thus, under favourable conditions, birds may remain within a given locality. In either case, expression of seasonal, photoperiod-driven migrations in nomadic migrants could function to prepare birds to move at times of the year when resource availability is likely to be changing, or to ensure that birds return to suitable breeding habitats following facultative movements (e.g. irruptions) to atypical areas [25,30]. Given that facultative migrations also occur at other times of the year, other cues must also play a critical role in triggering movements. Information about environmental conditions, such as food availability, either locally or at more distant sites, is likely also to be important in driving facultative migrations [25].

This study adds to a growing understanding that facultative migrants share more in common with obligate migratory birds than previously recognized. Our results provide clear evidence that the primary environmental cue that stimulates spring migration in obligate migrants, increasing day length, also triggers migratory preparations and behavioural readiness in a species with highly variable migratory movements. Given that other species of facultative nomadic migrants also seasonally express migratory physiology and/or behaviour in captivity in the absence of other likely migratory cues such as declining food availability [30,31], we suggest that the use of photoperiod as an initial predictive cue for migratory preparations, and potentially migration itself, may be a common feature across numerous nomadic species.

## Supplementary Material

Pine siskin photoperiod supplementary methods and analyses

## Data accessibility

The datasets as part of this study have been uploaded to Dryad: http://dx.doi.org/10.5061/dryad.6j90jp8 [68].
