# Supplementary material for: Increasing photoperiod stimulates the initiation of spring migratory behaviour and physiology in a facultative migrant, the pine siskin
Source: R Soc Open Sci. 2018 Aug 8;5(8):180876. doi: 10.1098/rsos.180876 (PMC6124035; doi:10.1098/rsos.180876)
Supplement: Pine siskin photoperiod supplementary methods and analyses [file rsos180876supp1.pdf]

Electronic Supplementary Material for: AR Robart, M McGuire, and HE Watts. Increasing photoperiod stimulates initiation of spring migratory behaviour and physiology in the facultative migrant, the pine siskin. Royal Society Open Science.

Material and Methods

(a) Animals

Pine siskins used during the Photoperiod experiment came from four capture sites in the western United States: Wrightwood, CA (May 2015; 34°21′N, 117°37′W); Randle, WA (August 2015; 46°18′N, 121°32′W); Jackson, WY (September 2015; 43°28′N, 110°49′W); and Eagle Point, OR (December 2015; 42°30′N, 122°49′W). Birds used during the Timing experiment came from a total of six capture sites in the western United States. In addition to birds captured at the Randle, WA; Jackson, WY; and Eagle Point, OR sites, birds were captured in Los Angeles, CA (March 2016; 34°07′N, 118°12′W); Cle Elum, WA (July 2016; 47°19′N, 120°39′W); and Leavenworth, WA (July 2016; 47°36′N, 120°50′W). Thirteen birds were subjects in both experiments. Birds were sexed by the presence of a brood patch or enlarged cloacal protuberance. In the absence of these features, sex was genetically determined from a blood sample (DNA Solutions, Miami, FL or the Moore Lab of Zoology at Occidental College, Los Angeles, CA). Birds were aged (as hatch year or after hatch year) using feather wear and moult and skull ossification [1].

(b) Flight muscle colour quantification

Flight muscle colour changes from dark maroon to light pink, with the change associated with increased lipid content in the muscle (M. Ramenofsky, personal communication). This

colour change is often observed with other physiological changes that are indicative of a migratory phenotype. We developed a non-invasive objective measure of colour and tested it against an alternative method of visually scoring colour using colour standards.

For the visual scoring method, we compared the bird's flight muscle colour to paint chips. Birds were scored as a "3" (the lightest category) if their muscle colour corresponded to the Benjamin Moore paint chip "1287" or lighter. Birds that had muscle colour that was best approximated by the Benjamin Moore paint chip "1281" were scored as a "2" (the intermediate category). Birds that had a darker, more maroon colour for their flight muscle than the 1281 paint chip were scored as a "1" (the darkest category).

For the objective, photographic method we used a Canon Powershot G11 to take RAW images, using standardized light settings and a grey colour standard with 18% reflectance (Figure S1). RAW image files were processed in ImageJ, using the Image Calibration and Analysis Toolbox software [2]. The right half of the flight muscle of each bird was selected, excluding any feathers, and the luminance quantified, as well as the red, blue, and green colour channels.

We used principal component analysis to analyse the relationship between luminance and all colour combinations that included the red channel. We saved the first principal component (PC) for all combinations (Table S1), each of which had an eigenvalue greater than 1 [3]. We then used linear mixed models to analyse the relationship between the PCs and the visually scored colour (Table S2). Mixed models included bird ID as a random effect (random intercepts); all analyses were performed in JMP Pro 12.0 (SAS Institute Inc., Cary, NC). We found that colour scored visually was significantly correlated with all PCs (Table S2), indicating a strong relationship between the two approaches (Figure S2). The Lum+R PC had the strongest

correlation with visually scored colour (Table S2), and this PC was used in the subsequent analysis of the effects of photoperiod treatment on flight muscle coloration.

(c) Statistical analyses

We used JMP Pro 13.0 (SAS Institute Inc., Cary, NC) for principal component analysis (PCA) to create a single measure of body condition that incorporated both mass and total fat score (furcular and abdominal). For both the Photoperiod and Timing experiments, we saved the first principal component (PC), which had an eigenvalue greater than 1 (Table S3) [3]. For the Timing experiment there was 1 bird that was an outlier for body condition (i.e., heavier/fatter). We conducted the analysis of body condition both with and without this bird; it did not qualitatively change the results and was left in the analysis.

References

1. Pyle P. 1997 *Identification Guide to North American Birds: Part I.* Bolinas, CA: Slate Creek Press.
2. Troscianko J, Stevens M. 2015 Image calibration and analysis toolbox - a free software suite for objectively measuring reflectance, colour and pattern. *Methods Ecol. Evol.* **6**, 1320–1331. (doi:10.1111/2041-210X.12439)
3. Norman GR, Streiner DL. 2008 *Biostatistics: the bare essentials*. Hamilton, Ontario: B.C. Decker Inc.

Table S1. Loading scores for muscle colour PCs. Lum refers to luminance, while R, B, and G correspond to the red, blue, and green colour channels, respectively.

PC	Lum+R	Lum+RB	Lum+RG	Lum+RBG
Lum	1.000	0.995	0.998	0.992
R	1.000	0.995	0.998	0.992
B	NA	0.980	NA	0.983
G	NA	NA	0.991	0.996
Eigenvalue	2.00	2.94	2.97	3.93
% Variation explained	100.00	98.08	99.09	98.33

Table S2. Linear mixed model results for relationship between PCs and visual colour score.

PC	*F* - value	df (numerator, denominator)	*p* - value
Lum+R	30.72	2, 346.3	< 0.0001
Lum+RB	23.89	2, 348.0	< 0.0001
Lum+RG	26.02	2, 349.9	< 0.0001
Lum+RBG	22.25	2, 351.6	< 0.0001

Table S3. Loading scores for body condition PCs for the Photoperiod and Timing experiments.

Body measure	Photoperiod	Timing
Mass	0.96	0.97
Total fats	0.96	0.97
Eigenvalue	1.86	1.89
% Variation explained	93.03	94.68

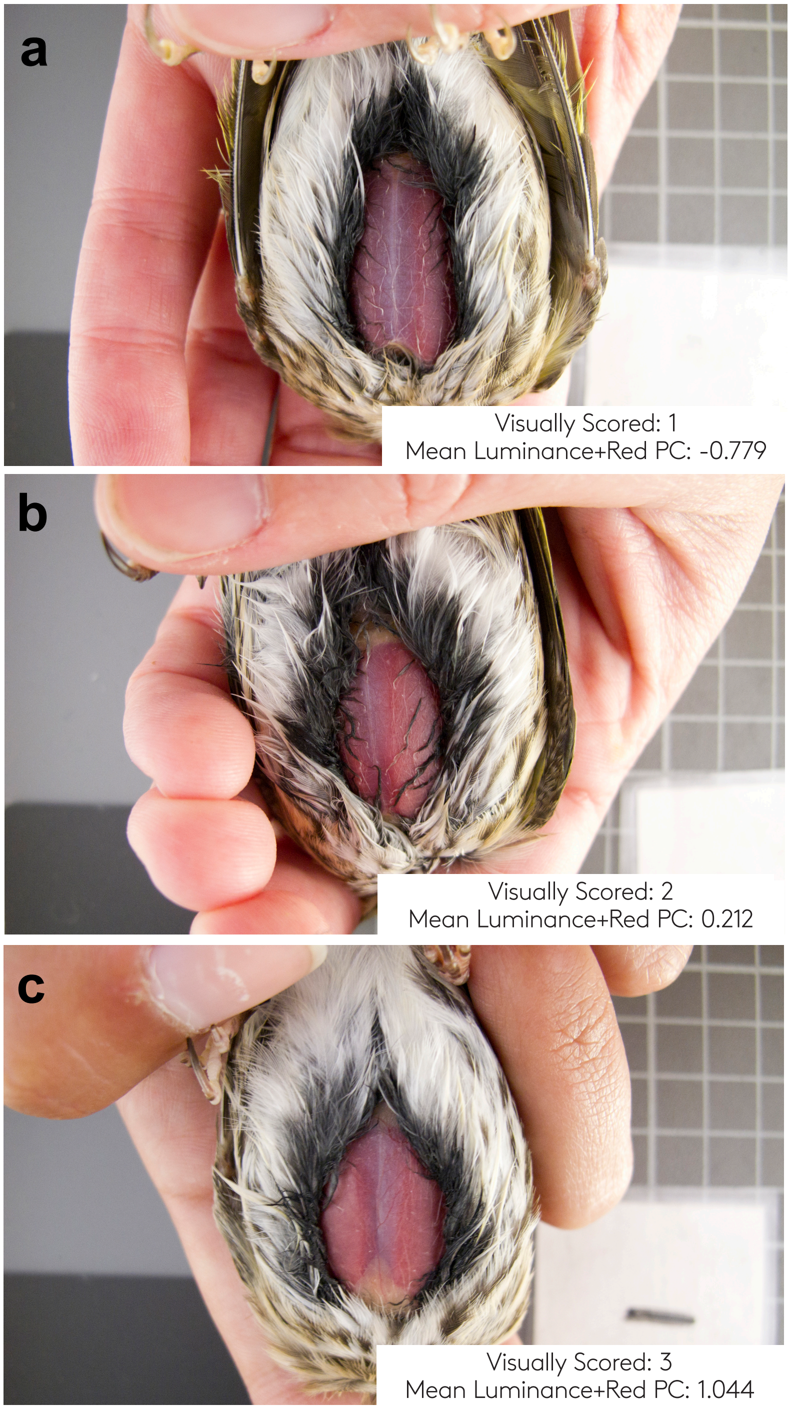

Figure S1. Examples of birds across a range of flight muscle colour scores. Birds are shown that were scored visually as being in the darkest (a) colour, intermediate (b) colour, and lightest (c) colour category. Values for both the visual score and the digital photographic analysis method are shown for each bird.

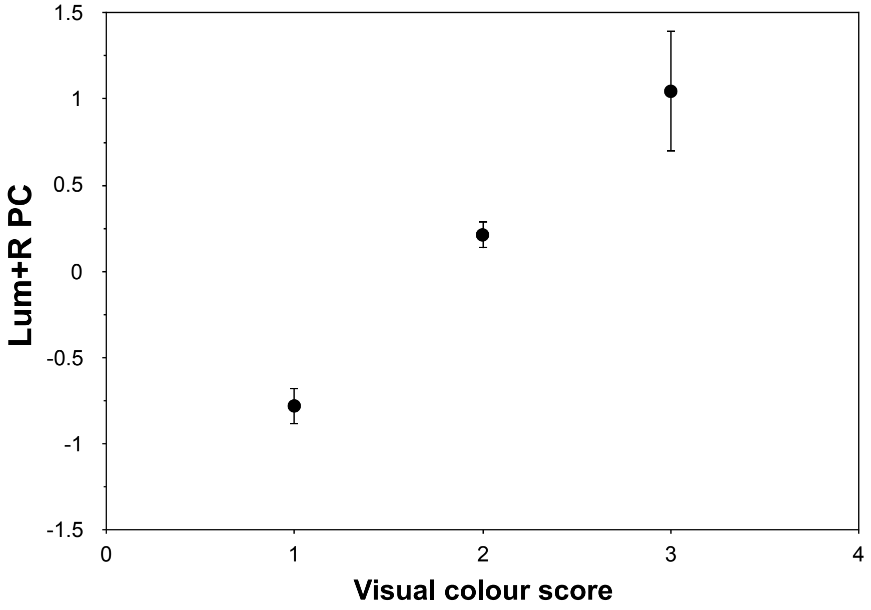

Figure S2. Relationship between visually scored colour and the Lum+R PC. Data points are means ± 1 SEM.

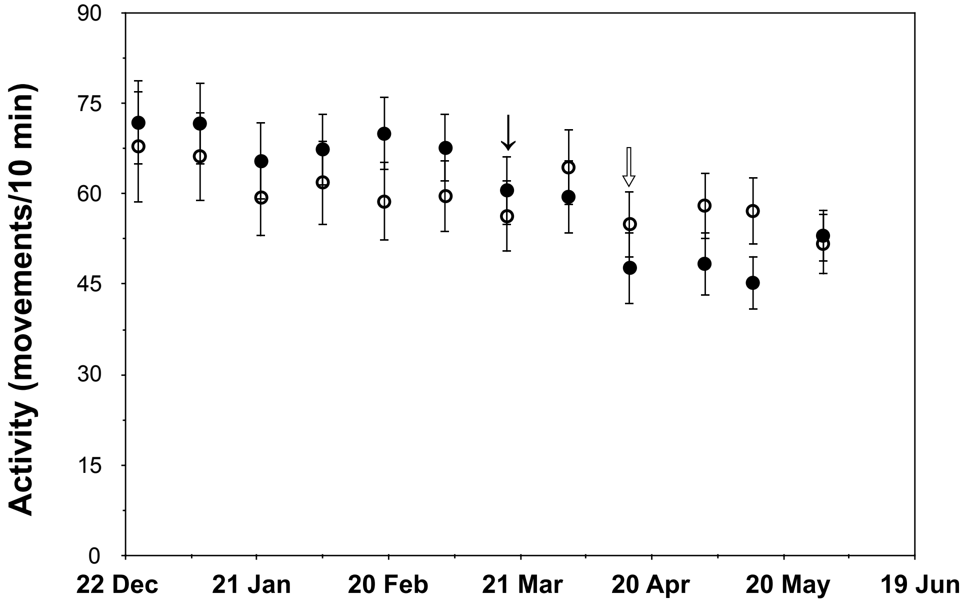

Figure S3. Diurnal locomotor activity of natural-day (filled circles and arrows) and short-day (open circles and arrows) birds during the Photoperiod experiment. Arrows indicate significant decrease in mean trait value, as indicated by change point analysis. Data points are means ± 1 SEM.

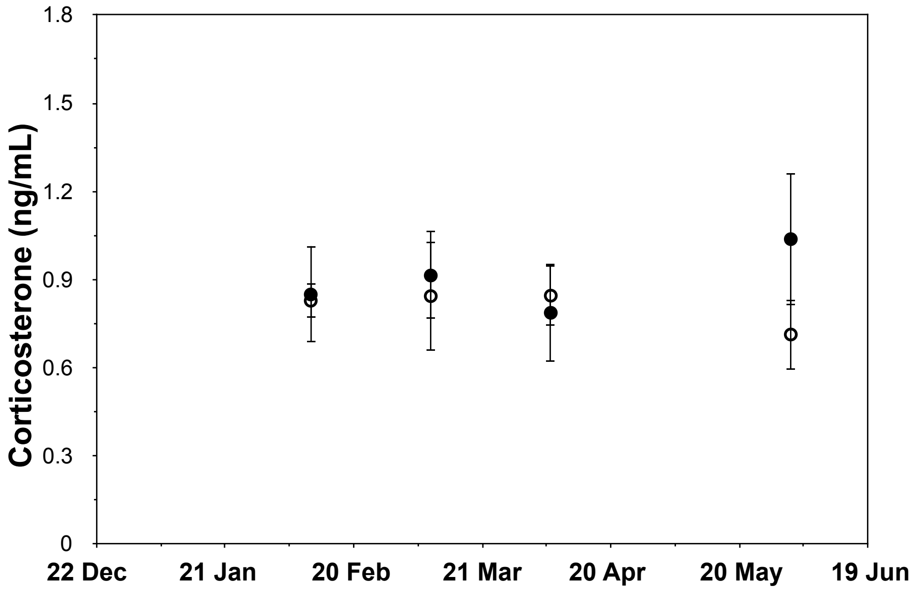

Figure S4. Corticosterone of natural-day (filled circles) and short-day (open circles) birds during the Photoperiod experiment. Data points are means ± 1 SEM.
